# Living with a Crucial Decision: A Qualitative Study of Parental Narratives Three Years after the Loss of Their Newborn in the NICU

**DOI:** 10.1371/journal.pone.0028633

**Published:** 2011-12-14

**Authors:** Laurence Caeymaex, Mario Speranza, Caroline Vasilescu, Claude Danan, Marie-Michèle Bourrat, Micheline Garel, Catherine Jousselme

**Affiliations:** 1 Centre Hospitalier Intercommunal de Creteil, Newborn Intensive Care Unit, Department of Research in Ethics EA 1610 ‘Études sur les Sciences et les Techniques’, (Studies on Science and Techniques) Paris-South University, Creteil, France; 2 Centre Hospitalier de Versailles. Service de Pédopsychiatrie, Le Chesnay, France; 3 INSERM U669, Université Paris-Sud and Université Paris Descartes, UMR-S0669, Paris, France; 4 Centre Hospitalier Universitaire Antoine Beclère (AP-HP), Clamart, France; 5 Service Hospitalier Universitaire de Limoges, Centre de la Mère et de l'Enfant, Limoges, France; 6 INSERM UMR S953, Epidemiological Reseach Unit on Perinatal Health and Women's and Children's Health, Villejuif, France; 7 Centre Hospitalier Universitaire de Gentilly, Fondation Vallée, INSERM U669, Université Paris-Sud, Paris, France; 8 Université Paris Descartes, UMR-S0669, Gentilly, France; Harvard Medical School, United States of America

## Abstract

**Background:**

The importance of involving parents in the end-of-life decision-making-process (EOL DMP) for their child in the neonatal intensive care unit (NICU) is recognised by ethical guidelines in numerous countries. However, studies exploring parents' opinions on the type of involvement report conflicting results. This study sought to explore parents' experience of the EOL DMP for their child in the NICU.

**Methods:**

The study used a retrospective longitudinal design with a qualitative analysis of parental experience 3 years after the death of their child in four NICUs in France. 53 face-to-face interviews and 80 telephone interviews were conducted with 164 individuals. Semi-structured interviews were conducted to explore how parents perceived their role in the decision process, what they valued about physicians' attitudes in this situation and whether their long-term emotional well being varied according to their perceived role in the EOL DMP.

**Findings:**

Qualitative analysis identified four types of perceived role in the DMP: shared, medical, informed parental decision, and no decision. Shared DM was the most appreciated by parents. Medical DM was experienced as positive only when it was associated with communication. Informed parental DM was associated with feelings of anxiousness and abandonment. The physicians' attitudes that were perceived as helpful in the long term were explicit sharing of responsibility, clear expression of staff preferences, and respectful care and language toward the child.

**Interpretation:**

Parents find it valuable to express their opinion in the EOL DMP of their child. Nonetheless, they do need continuous emotional support and an explicit share of the responsibility for the decision. As involvement preferences and associated feelings can vary, parents should be able to decide what role they want to play. However, our study suggests that fully autonomous decisions should be misadvised in these types of tragic choices.

## Introduction

Neonatal resuscitation makes it possible to treat newborns who because of severe damage from perinatal anoxia, congenital malformations or most often very preterm birth, require intervention to make the transition to extrauterine life and maturation. At the same time, epidemiological studies have shown various impairments in some of these survivors, findings that feed uncertainty about their future and oblige physicians to consider the utility and appropriateness of these interventions for each child. The answers to these questions about the future (very poor prognosis or intractable suffering) or present (no chance to survive, no-purpose situations) sometimes lead to a decision that life-sustaining treatments should no longer continue [1], [2], [3], [4]. Because newborns have no past and no known personality that would make it possible to determine their preferences, it is generally agreed that the best interests of the child should guide these decisions [5]. The distribution of roles between physicians and parents as surrogates of the child in this process has raised questions for many years [6], [7]. Parents are naturally called on to participate in the decision because of their parental authority and because they are the persons besides the child most affected by the decision's consequences.

The importance of involving parents is recognised by ethical guidelines in numerous countries [8], [9], [10], [11], [12]. However, results from studies exploring caregivers' and parents' opinions on this topic are far from unequivocal. Some caregivers prefer to exclude parents from explicit participation [13] or have them participate without making the final decision, to protect them from potential subsequent guilt [14], [15], [16]. Others, on the contrary, consider parents to be the best placed to make these decisions, at least in some cases [17]. On the other hand, some parents complain that they must live with the consequences of decisions made unilaterally by the caregivers [18], [19], [20]. Parents do not want to be excluded [21]; they frequently want to participate but not to decide, in view of the difficulty of the decision [20], . Cultural context can nonetheless influence the preferred type of involvement [24], [25].

A review of the literature shows that several aspects remain unclear: in studies involving parents, the notion of “taking part in a decision” seems to refer to diverse types of involvement, ranging from awareness of the decision to taking final responsibility for it. Other unclear issues include how end of life (EOL) decisions are taken, how parents construct and feel about those decisions, and what impact their type and content have on their future emotional well-being. This study sought to improve our knowledge of this area through an in-depth qualitative exploration of parents' experience of the EOL decision in the NICU. In particular, the study aimed to explore how parents described the decision making (DM), whether their feelings varied according to their perceived role in the decision process, the long-term impact of the experience in terms of guilt feelings and what they valued about physicians' attitudes in this situation.

## Methods

### Study population

The study included parents whose child died from 2002 through 2005 in one of 4 NICUs in different areas in France. All four units allowed unrestricted visiting for parents, and none had a specific protocol calling for family meetings for EOL decisions in clinical practice. Parents were contacted by letter about 2 years after the child's death and asked to participate in a face-to-face interview. A telephone interview was accepted as an alternative for those parents unavailable for the face-to-face interview. The letter described the study purpose (to help medical staff understand the experience of parents who had lost a child and thus improve their practices) and methods. The letter stated that if the parents did not respond, they would be called three weeks later. Parents were excluded if they did not speak French (n = 12), lived more than 100 km away from the interview site (n = 11) or if the child's physician objected to this contact (in most cases where civil or criminal legal proceedings were underway (n = 12) or when a parent had had a psychiatric disease requiring hospitalisation (major depressive disorder, bipolar disorder, psychoses, drug addiction) before the child's birth (n = 6).

### Data collection

In-depth face-to-face interviews lasted an average of 100 minutes. They were based on a thematic guide derived from a review of existing studies and three pilot interviews (not included in the final sample). A final version was established by the end of the 10th interview (see Appendix S1). Parents were asked to speak freely about their own perspectives, concerns and feelings about the child's history (pregnancy, delivery, NICU care, information about the baby's health, context of death) and about their emotional condition and life following the death up to the moment of the interview. Interviewers paid special attention to the parents' perceived involvement in the end-of-life decision-making process (EOL DMP), which is the focus of this paper. Telephone interviews were less structured and limited to topics spontaneously chosen by the parents. This procedure was employed for ethical reasons because telephone interviews do not allow interviewers to provide the direct emotional support to parents possible in face-to-face interviews.

Interviews were conducted by three skilled doctoral or master's level interviewers (CJ, MMB, CV) without clinical involvement in NICU care. Because these interviews could raise unanticipated emotional issues, in cases of distress, interactions were guided by the respondents' needs. Parents were able to ask questions and receive a referral to a mental health professional. Audio or video recording of the interviews (as chosen by the parents) and their full transcription made it possible to anonymise the data.

### Data analysis

Discourse analysis was used to study the data [26]. To take into account the subjective perspective of the qualitative method used in the study, the researchers disclosed their *a priori* opinions about the themes of interest, which varied from “parents should decide with the staff” to “parents should not be included because this would generate guilt feelings afterwards”. Separate identification and extraction of themes by both the principal investigator (LC) and a research psychologist (CV) optimised the validity of the results and helped find both known and new topics. Debating the discrepancies with a third skilled analyst (MG) until a consensus was reached ensured reliability. Attention was paid to the emergence of new themes, surprising findings, and contradictory results. We analysed our data within and between interviews [27] and discontinued data collection when saturation occurred (i.e., when new data consistently failed to contribute to refinement of the results) [27], [28]. The same methods were used to analyse data from telephone interviews.

Parental social and demographic data were collected at the interview. The children's medical history and other parental data were extracted from their hospital charts. Whenever available (92% of the charts), the description of the EOL DM reported in the charts was collected (LC). Statistical analyses were performed with the Statistical Package for Social Sciences (SPSS version 17).

### Ethics Statement

The study and the consent procedure were approved by the Medical Ethics Committee of the Intercommunal Hospital of Creteil, France. After we described the study to the parents, face-to-face respondents provided written informed consent, and telephone respondents oral informed consent. Results were collected in an anonymous database in accordance with French law and the regulations of the French Data Protection Authority.

## Results

Of 217 eligible families to whom letters were sent, 145 were reached; 12 declined to participate. Eighty families agreed to telephone interviews (37% of the eligible sample, 55% of the located families, 86 individual parents) and 53 to face-to-face interviews (24% of the eligible sample, 36% of the located families, 78 individual parents). Table 1 summarises the social and demographic characteristics of all respondent parents and the clinical characteristics of their infants. In all 164 individual parents of 139 infants participated. Among the face-to-face respondents, 25 pairs of parents were interviewed simultaneously. Interviews were conducted between 2005 and 2008. Participants' mean age was 33.9 (SD: 4.6) years at the time of the interview. Most were women (63%), and European (81%), with a minority of African parents. Half of the sample had high socioeconomic status (managerial and professional occupations) while 8.7% of the households included at least one unemployed parent. Interviews took place on average 2.8 years (±0.7) after the child's death. Comparison between respondents and non-respondents shows that non-respondent mothers were slightly younger (32.1+/−6.4 vs 28.5+/−5.8, t = −4.12, p<0.01) and more often unemployed (8.7% vs 21%, p<0.05). No other differences were observed for parents, or for any of the children's clinical characteristics.

**Table 1:** Description of the social and demographic characteristics of the respondent parents (N = 164) and their children (N = 139).

	Respondents
**Parental characteristics**	
Gender (females)	103 (63%)
Employed	150 (91%)
Managerial and professional occupations	79 (48%)
Skilled manual and non-manual occupations	71 (43%)
Maternal origin European	89 (81%)
Maternal age (mean)(years	32.1±6.4
**Parental contact with baby**	
No visit to the baby	18 (11%)
>2 visits to the baby	99 (60%)
**Child's characteristics**	
Gestational age (mean) (weeks of gestation)	31.2±5.9
Gestational age: preterm (<37 weeks)	99 (71%)
Sex: boy	84 (60%)
**Child's medical diagnosis**	
Systemic complication of prematurity (sepsis, ICH, NEC)	64 (46%)
Isolated CNS complication (cWMD, hydrocephaly)	17 (12%)
Peripartum anoxia, at term	40 (29%)
Congenital malformation/constitutional disease	18 (13%)
**Death preceded by decision**	93 (67%)
**Death without decision**	46 (33%)
**Medical status of the child at the time of final decision** *	
No chance to survive despite IC	25 (18%)
Theoretical chance to survive with IC, very poor prognosis	60 (43%)
Not dependent on IC but hopeless prognosis & severe suffering	8 (6%)
**Duration of life (median) (days)**	20.7±46.1
Duration of life <48 hours	30 (22%)
At least one parent present at death	98 (71%)

ICH: intracranial hemorrhage; NEC: necrotizing enterocolitis; cWMD: cystic white matter disease; IC: intensive care.

*Patients were classified according to their clinical status at the time of the EOL DM: those who had no chance to survive despite Intensive care (IC); those who had a theoretical chance to survive with IC but had a very poor prognosis; and those who were not dependent on IC but had a hopeless prognosis and severe suffering (Verhagen, 2007).

### Themes extracted from the interviews of parents' experience of the EOL DM event

Results are presented for face-to-face and telephone interviews together. However, perceived role in the EOL DM and related feelings are reported exclusively for face-to-face interviews because only data extracted from this source allowed us to classify parental role accurately: only in the face-to-face interviews did parents take the opportunity to extensively describe their involvement in the EOL DM. Telephone and face-to-face interviews were in basic agreement about their perceived role in the DMP and the emotions and guilt feelings related to it, although more parents expressed dissatisfaction during telephone interviews, especially about obstetrical care.

We did not observe any particular difference according to the study centre.

Extracted themes are illustrated with quotations (m refers to mother and f to father, numbers refer to the family; the letter “T” after the number refers to a telephone interview). The professional occupations of all the parents quoted in the paper are available (Table S1).

#### 1. Perceived role in the EOL DMP and related feelings

As mentioned above, here we describe results only from face-to-face interviews (N = 78). A third of the parents interviewed in person (N = 23) reported that *no decision* was made before the child died. In this case, the parent perceived that his/her child had died spontaneously without any discussion or any action by the staff (to withhold or withdraw treatments). “They *(the doctors) didn't even stop the machine; he died all alone; he fought for a day. He wanted to live. The machines were working as hard as possible. They couldn't do any more. So we were right to go all the way, to give him his chance*” (m45). All the other parents (N = 55) reported a decision with a specific perceived role in the EOL DMP. We identified three types of decisions: shared decision, which was the most frequent (N = 31), medical decision (N = 18) and informed parental decision (N = 6). (Table 2).

We defined the decision as *medical* when it was perceived as made by the physician without explicit parental involvement. “*As doctors, they considered that at some point it was necessary to decide to pull the plug. Therefore at that point, they suggested we all go to the bedside*” (m38). Medical decisions were the object of largely positive feelings. Many parents said that although they were not explicitly involved in the decision, they had reached the same conclusions as the medical team. Some spontaneously expressed relief that they did not have to decide, while others added that they found it impossible to express anything other than a desire for a healthy life for their baby: *“The doctor said to us: What do you want to do? We said to him: But you are the doctor, what would you do? Because what we want is for our child to be well”* (m29).
The decision was defined as *shared* when it was made after a discussion with the physicians, during which each person explained what mattered from their perspective and each agreed with the decision. *The shared decision* was appreciated overall because it allowed the parents to express themselves without having to decide alone: *“The doctor said to me, ‘your opinion is of course important, and your decision will be equally important, but you should know that the medical team also has an opinion and a decision.’ That was good. I said to myself, Thank god, it isn't me who has to decide. Because I had just been thinking what a real, total fright it would be to decide alone”* (m14). A majority pointed out the possibility of protection against guilt: “*I have the impression that (the doctors) act so that you have the impression that you are not making the decision yourself, so that you cannot hold it against yourself later*” (m32). All felt that confirmation by the doctors provided comfort and security: *“He said to us that the team would follow us in our decision, that he thought it was a good decision. That was great solace, because in fact it was our decision to make, and it was horrible to decide. At the time, it was great to be able to decide, that is, if a doctor had said to me, “We are deciding this”, it would have been unbearable for me. Here, it was difficult but the fact of having support, and hearing that … yes it did me a lot of good”* (m47). A minority of parents also stressed the importance of respect for their personal values: “*I think it's very important to be involved. You know there are people who have convictions which don't disappear, even though their child has no cerebral activity that would allow him a minimum of life*” (m38). None of these parents demanded greater involvement. Retrospective disagreement about the decision was found only once: a mother recently immigrated from Africa would have liked to oppose it. A minority of parents felt incapable of analysing the situation and found this sharing artificial: “*It has a supernatural feeling. You don't really realise anything. Me, I said yes to everything. We were acted on, not actors…*” (f17). One mother felt obliged to state her agreement, although she would have preferred to accept in silence. Guilt feelings and the weight of a “life or death” decision persisted for many parents within this shared DM group, despite the perceived involvement and support of the staff.
The decision was defined as *informed parental* when the parents considered the situation and made a decision without the doctors, after receiving full information about the medical data. *“They left us a full range of choices, based on our ethics, our morals, our religion”* (f21). The doctors applied the parental decision without influencing or reinforcing it. *Informed parental decision* was experienced negatively in most cases, largely because of a feeling of abandonment by the staff in a decision that involved the child's fate: “*They gave the choice to us, and it was difficult because they left us all alone, they left us really completely alone*” (f21). Only a minority experienced it positively, sure that they had made the right decision to relieve their child's suffering.

**Table 2:** Typology of perceived decision making based on the qualitative assessment.

Medical DM (N = 18)	Decision made by physician(s) No explicit parental involvement (tacit assent)
Shared DM (N = 31)	Discussion on the nature of the decision Exchange of relevant medical information (medically reasonable alternatives) Exchange of family values and preferences Parental choice about most appropriate decision Consensus reached with physicians
Informed parental DM (N = 6)	Medical facts given by physician Deliberation and final decision by parents No discussion of values
No decision (N = 23)	The child died before any decision was made concerning its treatments (modification or withdrawal,withhold of the treatments)

Analysis limited to face-to-face interviews.

On the whole, parents did not report the existence of an explicit discussion with the physicians on the distribution of roles in the DM. The parents most often accepted the role proposed by the doctor, without raising questions “*They made us choose, a little, to say: I thus ask you not to keep this living creature alive*” (f21).

Table 3 summarises the positive and negative feelings associated with the perceived role in the EOL DM.

**Table 3:** Positive and negative feelings related to the perceived role in the EOL-DM.

	Medical DM	Shared DM	Informed Parental DM
**Positive feelings**	Relief at avoiding an unacceptablechoice Avoidance of future guilt feelings	Relief of not having to decide aloneSatisfaction of dialogue	Empowerment Capacity to free the childfrom suffering Respect for personal values
**Negative feelings**	Disagreement about decision Absenceof dialogue Lack of confidence	Illusion: parental impossibility to express their viewpoint. Pretended parental agreement	Fear Solitude, abandonment DifficultyGuilt transgression

#### 2. Parental description of the EOL DM

This theme is extracted from data for the entire sample of parents (N = 164). Most parents described the decision as complex, neither chosen nor rational, and solitary. Complexity was linked to the effect it had on the family as a whole and to the sometimes contradictory interests involved, especially when based on the infant's future quality of life: “*It's selfish to say we are going to let her live for us. But it's also selfish to say that we are going to let her go to protect others*” (m38). It often seemed imposed, constrained by the facts: “*They (*the doctors*) came to tell us that she was going to die, at the same time, it was our choice — but what choice? As if you can talk about a choice. It was surrealistic for me*” (m49). Ambivalence was frequently suggested independently of the perceived role; although parents spoke about a “decision”, they didn't describe it as a positive choice: they decided something but did not will it to happen.

Most parents described having made the decision in a less than rational way, sometimes hurriedly or intuitively: *“I did not want to think about it, for me it was clear; I never even asked myself the question”* (m39). Emotions blocked many mothers - more frequently than fathers - in their ability to analyse the situation: “*At the time, all the emotions were different. I would have accepted a child with all the handicaps in the world, although I know very well today that that would not have been good for anyone*” (m44). For several parents the medical explanations were not sufficiently interpretable to serve as the basis for rational reflection: “*It was stories of percentages. Therefore in 50% of cases the children die of the side effects, and the 50% who remain, another 60% die. At the end, there was nothing. But I said, but what is she going to know about life?*” (m49).

Several stressed the difficulty of having to decide alone. *“The doctors say to us: ‘It's your choice. We are leaving the decision to you’. And finally, that is very very hard. I don't think it is a good thing”* (f21).

#### 3. Guilt feelings and interrogations in the long term

This theme reflects the current emotions related to the past decision. It is largely related to the parents' coping processes.

Guilt feelings related to the perceived role in the EOL DM are reported only for face-to-face interviews (N = 78), because we could accurately classify parental role only in those interviews. Of the parents interviewed in person, 48 of the 78 reported guilt feelings, 37 not related to the EOL DMP, 11 directly related to it. Most of the guilt feelings appeared to be independent of the decision. They were found in parents from all the groups, including those with no or medical decisions and were much more frequent among mothers than fathers. They could be related to pregnancy or premature delivery: “*Sometimes in moments of great distress, I can say I killed my daughter. But I think that the fact of being the mother, of having carried her, there is something else involved*” (m40). Other parents reported guilt feelings associated with the lack of a relationship with the baby during his or her short life, or to their absence at the moment of death or their helplessness, their inability to save the baby.

A smaller number of parents (11 parents out of the 78) expressed guilt feelings directly related to their role in the EOL DMP. Amongst these 11 parents, 3 perceived an informed parental decision and the remaining 8 a shared decision. A mother who perceived she had decided without the doctors, reported: “*We are the ones who said, then, on such a day, we stop. It is difficult for parents to tell themselves that they are not (well let's say) “killing” our child; it is that you stop, we stopped what was keeping her alive. You hate yourself*” (m27).

Persistent interrogations over the moral value of these past decisions were found among around half of the parents. This result was mostly observed in parents reporting an informed parental decision. Parents said they searched for arguments to make the decision acceptable and morally praiseworthy. However many found that to be difficult or impossible, especially those who thought that the decision was based on the child's future prognosis: “*Is it better to live with what we have now, or with an extremely handicapped child?*” (f5).
Three years after the decision, half of the parents said they couldn't accept the past decision: *“It's not a choice; it's something that you never admit”* (m27), many finally said they had to accept the decision, because its consequences were irreversible: “*After, you say to yourself, he is perhaps better off where he is than to live handicapped his whole life. In any case, you have to look at the positive side, or you will never get over it. You find reasons*” (f45). Some supported the decision afterwards by concluding that God or Nature had finally made the decision.

#### 4. Physicians' actions and attitudes perceived as helpful in making the decision and in coping with it afterwards

Most of the parents used the interview to transmit messages to medical staff about improving the decision-making experience. Several points emerged as most important in helping parents to cope with this decision afterwards: some involved the development of a trusting relationship with staff members, and others how doctors should be involved in the DMP.

Development of a trusting relationship:
Kind, non-judgemental involvement. The parents felt comforted in a protective, sympathetic and communicative ambience: *“They even asked me if I was hungry”* (f5). They appreciated dealing with the same caregivers the whole time: “*All 10 days, this paediatrician was there. She was really a person with whom we made decisions, choices, and she was there for us in the last seconds (…) She shared everything with us*” (f20). Care and attention to the baby were important: “*The whole team was great, especially during the care, the procedures, the precautions they took with him, always extreme consideration. That was important*” (f36). These factors gave them confidence in the staff and allowed some to express feelings that were difficult but determinative for the decision: “*I had a fear that I discussed with the doctors, in fact, I was afraid that she would live, to be honest. I said to myself that if they ever give us this, between quotations marks, “gift” of the child, alive, it is going to be a nightmare for the entire family*” (m25).
An interpersonal dialogue about the decision was praised; conversations with the doctor between humans on an equal footing made it possible to imagine the overall reasonableness of the choices. *“He explained that it was …I remember he said something: this isn't reasonable”* (f20). The family context and the realities of life had to be taken into account. “*The doctor left me the choice. He explained to me the risks of these choices. He told me, you already have a three-year-old daughter. He stayed in the context of our little family: for the child, for me, for my family. If something happens to you, who will take care of him? Very concrete questions*” (m114T).
Respectful language toward the child and the parents left a memory of the doctor's positive intentions: “*Doctor A always called the baby by her name: ‘Lena has very serious sequelae’. She was a person, not an ordinary case*” (m109T). Inversely, a disagreeable, barely involved attitude encouraged subsequent questions about the decision taken: “*This doctor, I don't ever want to see him again. When he told us that it was no longer legitimate to continue the resuscitation, he said it to us casually, without emotion, as if that happened to him every day. He was not warm. So, was he telling us the truth? That's a question*” (m98T).
An expert medical explanation, transmitted frankly, not necessarily in detail, allowed the parent to understand the situation: “*The doctor had explained the severity of the sequelae to us. He said to us, do you understand what that means? But obviously we did not know what that meant*” (m20). *T*he doctor should translate, repeat and refine the medical data without creating false hopes or using incomprehensible metaphors. Consistency among the professionals was reassuring.
Doctors' involvement in the DMP:
Parental desire for guidance in the DM varied amongst participants. More than half of the participants stated that the medical staff should express their opinions overtly and directively. These parents reported that they had felt overwhelmed by the situation (emergency, discovery of an unexpected malformation, or extreme prematurity) or by the exhaustion due to the baby's long hospital stay. Some mothers related this to their own weak health status in the post-partum. Other parents (approximately a quarter of the participants) preferred that the staff reveal its preference non-directively. Finally, a small minority reported that they did not need the staff opinion to decide.
The context of the decision (mother's health status, emergency, anticipation of the situation, the presence of a supportive partner) had a great impact on parental preferences for medical involvement. Overall, parental preferences were unrelated to their socioeconomical status: parents with the same occupation revealed different preferences, while different professions often were associated with the same preference.
Strong parental positions were in general not desired. For some, the decision was made by saying something to a given physician in an official setting. This gave them the impression of an action, a verbal action that could have been the cause of the child's death. Some had difficulty dealing with the fact that different doctors suggested different options: it made them feel obliged to take a position strongly and deliberately in favour of death. Many parents reported that doctors should explicitly involve themselves in the DM. *“Once we made our decision, it would have been best for the medical staff to be behind us, to tell us: you are right, this is what should be done, you've made the right choice”* (f21). The relief and security provided by the doctors' explicit position at least in supporting their choice was mentioned by many parents: “*Once we told them, they came to support our choice, saying, you've made the right decision… In fact they wanted to make us …not feel guilty*” (f18).

## Discussion

The objective of this study was to obtain a detailed qualitative description of the EOL DM as experienced by parents whose child died in a NICU three years before. To our knowledge, this is the first large study to use a detailed assessment of the perceived role played by each parent, making it possible to compare theoretical assumptions about DM with actual real-life experiences.

Results from data analysis of parental narratives identified three types of EOL DMP, in accordance with the current literature [29]: shared, medical and informed parental DM. Each type was associated with specific feelings afterwards. Overall, our study shows that the EOL decision is always complex, but often not really a choice for parents or rather, when the child is moribund, it is a Hobson's choice: if death can be prevented the step is nonetheless difficult to justify. Parents are tempted to flee such a stressful situation by making intuitive or rushed decisions, as shown in other stressful situations [30]. Moreover, decisions are perceived as complex because they involve contradictory interests, making it difficult to define the child's best interests clearly.

The complex nature of such decisions affects how parents experience their involvement in the DM. Most parents explicitly preferred DM that they perceived as shared, which appears to offer a balance between the active position that parents seek and their fear of being wrong and overwhelmed by the future weight of responsibility. Like others [31], we have observed how explicit medical support comforts parents in their choices even years later.

Many parents however accept that doctors make the decision. In this case some parents stated their preferences and implicitly left to doctors the duty to make a decision in their place, as in a “doctor-as-agent” model [32]. Others gave their assent to decisions already made by the team that they found appropriate, which allowed them to have their choice followed while avoiding responsibility for it. Finally, a small number of parents reported a decision made on their own without the doctors. The rarity of this autonomous decision might be related to the cultural context of the study: in France most neonatologists believe that parents in the NICU should not be required, or even allowed, to make the EOL decision alone [33], [34]. Although some could accept it, the majority strongly criticised precisely that aspect, being left alone to make the decision. They perceived it as isolation and abandonment. This result is one response to the question raised by Orfali [19] about whether feelings of abandonment are linked to parental DM itself, or to the lack of genuine caring relationships and “emotional work” by the caregivers [35]. According to our data, parents need doctors to provide a supportive presence and to clarify the issues at stake. Those actions are necessary but not sufficient: it is at the moment of the decision that the presence and position of the doctor become essential. Parents look for personal involvement from the doctor of the type suggested in patient-centred medicine [36] and a joint interest in seeking what is best for the child within the family. It is the perception of this sharing, of a “moral community” [6] that helps the parents to invest themselves in the search for the best decision, to think out loud without feeling judged. In the long term, this trusting environment reinforces the validity of the decision. On the contrary, in cases where parents perceived that they had decided without medical involvement, they often felt that the responsibility of having made a “life or death” decision was equivalent to a transgression: it belongs to God, nature, fate or possibly to the doctors but not to them. In these situations, feelings of guilt related to the decision making and persistent questions about the moral value of the past decision, might be more intense. This is in agreement with the findings of Botti and colleagues who showed that perceived personal responsibility for making tragic decisions generates more negative feelings than having the same choices externally made [37]. However, we also found interrogations about the decision in parents of the other groups, suggesting that other factors (such as personality or the sense of causality associated with the premature birth) are involved, as others have suggested [24]. Professionals try to anticipate and adjust to parental preferences for medical involvement in the DM. On this point, our study suggests that the context (mother's health status, emergency, anticipation of the situation, the presence of a supportive partner and the overall emotional climate created by the medical staff) in which the decision takes place weighs more than objective factors such as parents' socioeconomic status. We can suppose that in such extreme situations, individual social group differences tend to blur [35].

Some limitations of the current study must be taken into consideration when interpreting the findings.

First, there might be a bias linked to perceived role in DM if the individual perception does not correspond to what really happened. However, when data about the DM were available in the medical files, they were highly correlated with classifications of direct parental assessment. The discrepancy between parental and chart reports was less than 10%.

Second, the difference in handling in-person and telephone interviews might have induced a bias in the data. It is probable that the method of recruitment, excluding parents involved in malpractice suits, might have minimised the truly negative perceptions.

Third, the limited response rate of the study should be mentioned. Nonetheless, this response rate is relatively high for vulnerable samples, especially long after the death of a child [38]. Moreover, differences between participants and non-respondents for parents' socioeconomic status and for the child's medical history were relatively small. Finally, this study took place 3 years after the child's death. It did not develop the long-term outcome of EOL DMP on the family structure or on the parents' social situation. Future studies could specifically investigate these topics, at different points in time and in relation to the coping strategies used by parents.

In conclusion, many parents find it valuable to express their opinion in the EOL DMP of their child. Nonetheless, they do need continuous emotional support, a trusting relationship, and an explicit share of the responsibility for this decision. As involvement preferences can vary, real shared DM should also enable parents to decide the role they want to play in this crucial situation. It should be borne in mind that in these types of tragic choices, parents' subsequent coping would be aided by physicians' recommendations that the parents not take a fully autonomous decision. Deeper thoughts about the child's best interests might help to put these decisions into a clearer context. Concepts associated with communication and patient-centred medicine and parental insights could serve as a basis for training NICU professionals.

## Supporting Information

Table S1 **Occupations of parents quoted in the paper.**
(DOC)

Appendix S1 **Outline of parent interviews.**
(DOC)
