# Supplementary material for: Living with a Crucial Decision: A Qualitative Study of Parental Narratives Three Years after the Loss of Their Newborn in the NICU
Source: PLoS One. 2011 Dec 14;6(12):e28633. doi: 10.1371/journal.pone.0028633 (PMC3237456; doi:10.1371/journal.pone.0028633)
Supplement: Table S1 — Occupations of parents quoted in the paper. (DOC) [file pone.0028633.s001.doc]

| **Table S1. Occupations of parents quoted in the paper** | | |
| --- | --- | --- |
| **Participant’s code** | **Occupation** | |
|  |  | |
| **Fathers** | | |
| f5 | | Technician |
| f17 | | School teacher |
| f18 | | Transport worker |
| f20 | | Secretary |
| f21 | | Statistician, engineer |
| f23 | | Information technician |
| f36 | | Sales engineer, MBA |
| f45 | | Salesman |
|  | |  |
| **Mothers** | | |
| m14 | | Communications director |
| m20 | | Day care worker |
| m25 | | Sales engineer |
| m27 | | Nurse |
| m29 | | Medical secretary |
| m32 | | Not employed |
| m38 | | Maître d’hôtel |
| m39 | | Optician |
| m40 | | Social worker |
| m44 | | Dental assistant |
| m45 | | Secretary |
| m47 | | Sales engineer, MBA |
| m49 | | Secretary |
| m98T | | Bank employee |
| m109T | | Civil servant, city hall |
| m114T | | Administrative worker |
| m132T | | Administrative worker |
