# Supplementary material for: Living with a Crucial Decision: A Qualitative Study of Parental Narratives Three Years after the Loss of Their Newborn in the NICU
Source: PLoS One. 2011 Dec 14;6(12):e28633. doi: 10.1371/journal.pone.0028633 (PMC3237456; doi:10.1371/journal.pone.0028633)
Supplement: Appendix S1 — Outline of parent interviews. (DOC) [file pone.0028633.s002.doc]

**Appendix. Outline of parent interviews**

At the beginning of the interview, the interviewer suggested that they tell the entire story as a narrative covering everything from the pregnancy until the moment of the interview.

**Sample questions (to be asked if this information does not appear spontaneously in the parents' account)**

At what moment did you realise or understand that your child was going to die? In what circumstances?

Could you tell us how the decision to reorientate care was reached ?

Do you feel able to describe your child's death to me?

Who was there? Was the family there? What medical team personnel were present?

Who did you miss most at that time?

What did you miss most in the period before your child died?

Who would have been most useful to you at that moment?

How did you spend the last minutes you had with your child?

Did anything during care in the intensive care unit shock or hurt you?

In the intensive care unit, what did you find shocking, and what helpful?

After your child's death, did you see again one of the doctors or nurses, or a psychologist or psychiatrist, or parents' associations?

*For those who have other children:*

How did you tell them about the baby's death at that time? What were their reactions?

And for the baby after all that?

Did you keep any pictures or videos of the baby who died? Do you look at them? Under what conditions?

Did the baby's death lead to any changes in your life (in the family, in general etc…)?

Can you tell me some adjectives that characterize in your mind the baby who died?

During all the care in the ICU, can you tell me what made you able to feel that you were the parents of the baby you lost?

What would you like to say to neonatal resuscitation and intensive care teams who manage children in the situation your baby was in?

What would you like to say to the familles living through the events that you experienced?

How and why did you decide to agree to come share these memories during an interview?
